# Bibliographical Notices

**Published:** 1856-05

**Authors:** 


### BIBLIOGRAPHICAL NOTICES.

*The Obstetric Memoirs and Contributions of* JAMES Y. SIMPSON, M. D., F. R. S. E., &c. Edited by W. O. Priestly, M. D., Edinburgh, &c., and Horatio R. Storer, M. D., Boston, U. S., &c. Vol. i.; Philadelphia, J. B. Lippincott, & Co., 1855.

Though many, distinguished by their literary and professional attainments, have vigorously opposed Dr. Simpson's theories and modes of practice, yet we believe that no one has ever disputed his talents, his zeal, or the rare ingenuity, originality and untiring energy by which he has placed himself in the front rank, if not at the very head of obstetrical science. No candid man can deny to him this honor, for he combines all the very rarest requirements of a great medical and surgical career; fearless, yet prudent, quick and sagacious, his reasoning, though seeming to border on temerity, has generally been fully justified and supported by the results of his practice. Thus impressed, we need hardly add that we hold ourselves and the profession in the United States as under great obligations to the gentlemen who have with so much skill and care collected and arranged the varied and interesting contents of this volume.

Dr. Simpson's obstetrical contributions, heretofore scattered through medical journals and transactions, have not yet, we think, in this country, received the careful investigation which they deserve. Particular articles, when wanted for reference, could not be readily found; hence the practical proof of the value of some of his suggestions is in a great measure still wanting. The work in question is therefore a most valuable addition to our obstetrical literature, and we have no doubt will be eagerly sought after by all reading medical men in the United States.

We will mention that the memoir on the construction and uses of the uterine sound, and its great value in the correct diagnosis of intra-pelvic diseases, is ably and clearly written, and proves that the author had given great care and attention to the subject. The majority of the profession is now fully impressed with its great usefulness; but the views of the originator on the subject, and his various illustrations of the manifold advantages which it affords to the investigator, will, we have no doubt, make it a very popular auxiliary to our other modes of determining the nature and extent of many obscure uterine diseases.

As we have heard it more than once objected to the use of this instrument, that it was dangerous on account of the irritation and even inflammation likely to be occasioned in so delicate and important an organ as the uterus, and having ourselves no experience in its use, we give Dr. Simpson's words on the subject of its effects:

"The degree of uneasiness felt by the patient during the passage of the instrument is in general very trifling, and not more, if so much, as is felt on passing the catheter along the urethra of the female, and certainly not by any means nearly so great as in using the sound or bougie in the male. In a few cases I have seen it, like the passing of the sound in the male, produce a feeling of sickness and nausea. In the healthy state, however, of the organ, the lining membrane of the uterus does not in fact appear to be more sensitive than that of the vagina, so that the existence of any true and actual pain in making the examination with the Bougie, is to be considered so far anormal, that it is generally, as we shall afterwards see, indicative of the existence of some morbid state or other of the part or parts with which the instrument is at the time in contact."

We have nothing to say against the use of such an instrument in proper hands, when, of course, it will never be used unnecessarily; but we fear that, like the speculum, its application will be terribly abused. Women are easily persuaded by a plausible charlatan, that they are laboring under some uterine disease, and in their terror they submit to manipulations which are more useful to the rascally operator's pocket, than to the libelled organ in question. We know a case where one of these respectable individuals recommended the introduction of the speculum to a young girl scarcely nineteen years of age, for a simple leucorrhœa. The family very properly dismissed him, and sent for a respectable physician of our acquaintance, who cured the so-called ulceration of the womb by the simplest means imaginable. The ease with which, as Dr. Simpson assures us, his sound can be introduced, will render it a capital travelling companion to the speculum.

In this brief notice it is impossible even to name a fraction of the various subjects discussed; but we naturally turn from the uterine sound to one of a kindred nature, though we think of far more doubtful utility—we mean the intra-uterine pessary—for the modern application of which we are indebted to the extraordinary zeal and ingenuity with which our accomplished author appropriates and modifies every thing which can in any way contribute its assistance to his professional efforts. The instrument, its construction and application, are well described, but the keeping it in situ, as allowed by the Doctor himself, appears to be a somewhat difficult and troublesome affair; the external wire apparatus, bent over the pubis and supporting the internal portion of the instrument, by means of curvilinear tubes, locks, &c., &c., appears to us to be far too complicated to be of general practical utility. We have no doubt but that there may occur some cases of badly retroverted uteri, which may require such complicated machinery to keep them in place, though we have never been so unfortunate or fortunate, according to different tastes, as to meet with such. Dr. Lee, of London, made a savage onslaught upon this instrument, but the inventor clings to it very naturally with parental fondness. Its use will, of course, find favorers, and a sufficient experience, only to be gained by time, will doubtless ultimately lead to a just professional verdict on its merits.*

The article on the detection and treatment of intra-uterine polypi, is one of the most interesting in the collection. In cases of frequently recurring hæmorrhage, where the os uteri remains closed and the diagnosis doubtful, Dr. Simpson tells us that he is in the habit of opening the os by means of sponge tents, and has thereby frequently succeeded in discovering and removing intra-uterine polypi, which certainly, from the exhausted and anæmic condition of the patients, must have led to fatal consequences. He describes minutely the mode of preparing and introducing these tents. A well made tent generally takes from twenty to thirty hours to expand to its full extent, and dilates to four or five times the diameter it presented in its original

* In a late number of the London Lancet, (March 29th,) Dr. Henry Bennett states that "several fatal cases have occurred in England, and in Paris seven deaths from the use of the intra-uterine pessary have been published—the one of M. Amussat, which occurred in 1826, and six recent cases." Five of these were from acute peritonitis, one from secondary pelvic abscess.—Ed.

compressed state. One tent is often sufficient for all the purposes desired; but if not, a larger one is introduced, and so on until the womb is completely opened, when the operator can, with his finger, ascertain the condition of the whole internal surface of the organ.

Small, vesicular polypi, Dr. S. has often removed by avulsion; sometimes we know that they may be detached by the finger. The author prefers excision by knife or scissors to the ligature in all cases of large pediculated tumors. He makes light of the dangers of hæmorrhage, which have been advanced as an objection to this mode of practice, and says that it can always be controlled by the tampon. Indeed, after operating he always introduces one, which he suffers to remain twenty-four hours.

The process of excision is certainly rapid—the affair of a few minutes. The deligation of a uterine polypus is rather a succession of operations than one, and if the ligature be long in doing its work, as is sometimes the case, we may have great constitutional irritation to contend with, besides the horrible fœtor of a slowly putrifying mass, adding greatly to the misery and apprehension of the patient. "After an interval," observes Dr. Churchill, "varying from six days to three weeks, the canula will be found loose in the vagina and the stalk of the polypus severed.

Dr. Simpson makes the following remark on the subject:

"Those authors who have written in favor of deligation, usually quote one solitary case of death from hæmorrhage after excision, recorded by Zacutus Luzitanus, in the seventeenth century. It was an instance of the fact that the amount of attendant hæmorrhage is not regulated by thy mere size of the polypus; for in the case in question it is stated that the amputated polypus was not larger than an almond. In this instance the operation was performed by an empiric, and no plug or other means for arresting the hæmorrhage appear to have been employed. The patient died, not so much from the operation as from neglect of all proper means for arresting the hæmorrhage resulting from it."

"But the principal danger to health and life in this, as after other surgical operations, is the danger of phlebitis and surgical fever. Is such a consequence more likely to follow upon the instantaneous resection of the peduncle of a polypus and its subsequent immediate removal, or is it more likely to supervene upon the slow process of disjunctive ulceration being set up in the stalk of the polypus by the ligature, while the gangrenous mass itself is left decomposing in the cavity of the vagina?"

The article on the subject of the operation of ovariotomy, is not written by Dr. Simpson, but is a report by the editor of the Edinburgh Monthly Journal, of his opinion of that proceeding, delivered before the Medico-Chirugical Society of Edinburgh, on the 17th of December, 1845, in which it is stated that Dr. S. expressed his conviction that he believed ovariotomy quite unjustifiable in many of the cases in which it had been had recourse to, while at the same time, he proved by statistical tables, that it is not more fatal than other capital operations.

The report likewise informs us, that the Doctor is of opinion that by means of the uterine sound and a careful manipulation, a correct diagnosis between ovarian and fibrous tumors of the uterus may always be attained, thus removing one of the great objections to the undertaking. He is likewise represented as recommending the use of exploratory needles, to ascertain in doubtful cases the nature of the contents of these tumors; that he believed the chief difficulty at this moment is assuredly that of discovering the existence or not of adhesion of the tumors by false membranes, their extent, &c., that if this point could by any means be cleared up, it would remove one of the great, perhaps the greatest, existing objections to ovariotomy.

But of the mode of accomplishing this we are left totally in the dark. The author, we are told, assures his audience of what in all probility the profession in Edinburgh knew before, viz: that modern pathology has made wonderful advances, and that physical diagnosis may, at some future period, help us out of the difficulty; that some of our domestic animals enjoyed a remarkable impunity from the coarse operations of spaying; that, as proved by the Cæsarean section on the continent, the peritoneum took more kindly to cutting than was at one time supposed, but still that extreme dread of all such abdominal surgery was, and probably is, the prevailing idea. This last statement is to us, we confess, extremely gratifying, and we are pleased to be able to record the fact, that this eminent authority thinks it somewhat cruel and even discreditable to cut a woman open on an uncertainty.

Turning as an alternative for craniotomy and the long forceps in deformity of the brim of the pelvis, is a long and elaborate essay, originally presented by the author to his pupils, in 1850. It is admirably illustrated by cases and diagrams, and the mechanical advantages of the practice clearly explained; but as we could not pretend to give any idea of the merits of the article without making long extracts, for which we have not room, and as it has been long before the profession, we satisfy ourselves by recommending it as a study to those who may be interested.

Of the long and elaborate treatise on placenta previa, we are not called upon to speak; the subject has long been in consideration by the profession, and Dr. Trask's admirable prize essay has given us the results of all modes of treatment, in copious and carefully prepared statistical tables. In reviewing these, it appears, that in certain cases, that is, when the hemorrhage is great and the close and rigid contraction of the os uteri forbids immediate delivery by turning, the propriety of Dr. Simpson's proposition to detach the whole placenta is sustained.

To conclude, we have no hesitation in asserting, that we think this one of the most interesting and instructive books we have ever read; and though the author differs from many preconceived notions of his professional confréres, yet there is preserved throughout every article, a tone of courtesy and respect when speaking of the opinion of contemporaries, which makes the reader entirely forget that some of these gentlemen were and are his fierce opponents. We cannot but commend the spirit which dictated such a course; and cannot help expressing a regret that the Boston Editor should, in his preface, have forgotten, in his enthusiasm for Dr. Simpson's cause, the precept and even the expressed request of his instructor, by impugning with unseemly bitterness the motives which induced Dr. Lee, of London, to enter the lists against his friend; Dr. Simpson had the sagacity, as well as the modesty, to see, that a sarcastic depreciation of others is not the true path to the temple of fame, and even if he felt that some one had opposed him with unfairness, his admirable motto was, "let bygones be bygones." We think his well wishers would have better served his dignity by having the good taste to say, "so be it."

*Physical Exploration and Diagnosis of Diseases affecting the Respiratory Organs.* By AUSTIN FLINT, M.D., Professor of the Theory and Practice of Medicine in the University of Louisville, etc.

In noticing any new volume on a subject upon which numerous works have been published, it is perhaps best to suffer the author to appear in his own introduction:

"The few special treatises which have more recently appeared, are mostly designed as manuals for the medical student." . . . . .

"My aim has been to supply what appears to me a desideratum, viz., a work limited to diseases affecting the respiratory organs, treating *in extenso*, and almost exclusively, of the principles and practice of physical exploration as applied to the diagnosis of these affections. So much, briefly, for the motives and objects which have prompted the undertaking."

In a treatise of this kind, of course, much must have been gathered from the writings of others; yet the work before us is not deficient, either in original thought or observation. We proceed to notice those subjects which seem to have been specially studied by Dr. Flint.

*Percussion.*—The appreciation of the results of percussion in disease must be based upon our knowledge of the sounds a healthy chest yields when struck. The fact that we are aware, that such, or such a part of the chest, ordinarily sounds clear, or dull on percussion, does not aid us in individual cases; it is the comparison between the two sides on which we base, or, at least, ought to base our diagnostic conclusions. To determine, then, to what extent disparities exist in health between the two sides of the chest, must be a matter of great practical importance; and this is one of the points to which Dr. Flint seems to have paid particular attention.

In the post-clavicular regions, percussion does not produce uniform results, on account of the impossibility of applying the finger, or the plessimeter, equally on both sides. This should, therefore, make us very careful in attributing any slight differences in this region to a morbid condition. Over the clavicles a disparity frequently exists in healthy chests, and there also slight differences should not be considered as abnormal. In the infra-clavicular region, Dr. Flint detected a very frequent dissimilarity between the two sides. The sound, on percussion, had generally a clearer or, as it is called, a more "vesicular" character on the left side, and was lower in pitch. At the summit of the chest, behind—the *scapular* region,—there was less disparity of sound, although even here the pitch was higher on the right than on the left side. The important bearing of these observations is obvious, especially for the recognition of tubercular disease. We should feel, however, more confidence in the results, if the observations had been made on a more extended scale than the number indicated (20) would imply; as, moreover, in eight out of these the percussion sound was, "in all respects, equal on the two sides in the region first indicated." In several other regions of the chest equal diversities were observed between the two sides. The whole chapter, indeed, involving the differences in healthy percussion, we recommend as worthy of attention.

Percussion, as applied to disease, Dr. Flint studies under four heads: 1. Exaggerated vesicular resonance. 2. Diminished vesicular resonance. 3. Absence of resonance or flatness. 4. Tympanitic resonance. This latter resonance is almost always accompanied by a rise in the pitch of the sound. More importance is indeed attached throughout by Dr. Flint, to the differences in pitch, than is done by most authors. Skoda declares that changes in this respect are not entitled to much value in practice. Walshe mentions the differences in pitch and illustrates them by the difference of the heart and liver sound *in situ*, yet he attributes to the pitch of the sound but little diagnostic import. The author before us believes the pitch of sound to have been much neglected.

"A variation in pitch by one who has what is called a 'musical ear,' is more easily recognized, than a slight disparity in the amount of resonance; and in some instances the former may be distinguishable without difficulty, when the latter is inappreciable. In cases, therefore, of suspected tuberculosis, it is important to compare the sounds on the two sides as if they were musical notes, in order to determine whether they are in unison, or differ in their diatonic relation to each other. A difference in pitch may then be the only discoverable evidence of dissimilarity, and, in connection with other signs and symptoms, may be entitled to considerable weight in the diagnosis. The importance of attention to the pitch of percussion-sounds, with a view to greater nicety and accuracy of discrimination, seems to me not to have been sufficiently appreciated by most writers on the subject of physical exploration."

Under the head of the tympanitic sound are embraced the amphoric and the cracked metal sound. This latter is almost always associated with a cavity communicating with a bronchus. Dr. Stokes, in his work on Diseases of the Chest, mentions nevertheless the fact of its occurrence in cases of bronchitis with effusion. We have ourselves seen this sound produced in children by a strong and rapid percussion without any evidences of consolidated or excavated tissue existing. Dr. Flint cites two instances of this sound without any cavity having been found after death.

*Auscultation* is undoubtedly a more popular method of physical exploration than percussion. It requires less skill to hear sounds, than tact and practice to produce them. The sounds, on application of the ear, are with a little attention soon recognized; their mechanism and the causes of their production are the points that baffle. Auscultation, when its object is to isolate sounds, is best performed by means of a stethoscope. Of stethoscopes, there is every variety of shape and form. The flexible stethoscope recently invented by Dr. Cammann, of New York, has been extensively used by Dr. Flint, and he expresses himself much satisfied with the result:

"In making trial of the instrument, I have found it more difficult to institute comparisons as regards quality and pitch of sound than with the ear alone, or the ordinary stethoscope; but with reference to differences of intensity, and in rhythm, it admits of a wider application than the common modes. It renders distinctly audible, also, morbid sounds in some instances in which they are too obscure to be studied satisfactorily without its aid. For comparison of the two sides of the chest, as respects the resonance produced by the act of speaking, it is exceedingly well adapted." (p. 131.)

"In cases of suspected tuberculosis, in which tuberculous deposits are either wanting, or are small and disseminated, by means of this stethoscope a closer comparison of the respiratory sounds can be made than with the ordinary cylinder or the naked ear. A disparity, therefore, on the one hand, is in some instances rendered appreciable which otherwise would not be discovered; and, on the other hand, the absence of a disparity, and the completeness of the normal characters, are more satisfactorily determined than is always practicable without this improved means of auscultatory exploration. It enables the auscultator to study the characters of the respiration in some cases in which it is so feeble as to be with difficulty appreciated by the ordinary cylinder or by immediate auscultation. Its usefulness in cases in which it is desirable to make nice comparisons with respect to vocal phenomena, is not less than in examinations with reference to respiratory sounds." (p. 485.)

The greatest draw-back we have noticed to its use is the intensity it gives to any sound produced by the friction of the stethoscope against the clothing, or even against the walls of the chest; a circumstance which undoubtedly interferes with an accurate appreciation of the intra-thoracic murmurs.

The sounds of respiration require as much as the sounds of percussion, to be first studied in the healthy chests. They are divided by some authors according to their character; by others, according to the anatomical situation of their production. Dr. Flint distinguishes a tracheal, a bronchial and a vesicular respiration. The tracheal is tubular in quality, high in pitch; the inspiration and expiration are separated by an interval. In the larynx, according to Barth and Roger, its character is altered; such is not, the opinion of Dr. Flint. The bronchial murmur is heard near the sterno-clavicular articulation and in the interscapular space; it is high in pitch and consists mostly of an inspiratory and expiratory sound. In 24 examinations it existed anteriorly in the act of inspiration only, twelve times; its intensity (p. 143) is rather greater on the right side.

The vesicular respiration is mainly an inspiratory murmur; the expiration is absent in about one-third of the cases. It is lower in pitch than the inspiration, always shorter than this sound, and feebly tubular or blowing.

The vesicular sound is, as first pointed out by Dr. Gerhard, of unequal character on the two sides of the chest. Dr. Flint has found in the infra-clavicular, or "tubercular regions," if we may be permitted to use that expression, the inspiration on the right side less intense and less markedly vesicular, although of a higher pitch than on the left side; the expiration more prolonged and intense, and of a higher pitch than the sound of inspiration.

*Comparison of Right and Left Infra-clavicular Regions. Whole number of examinations twenty-four.*

INSPIRATORY SOUND.

| *Right.* | *Left.* |
|---|---|
| Greater intensity in one case. | Greater intensity in 15 cases. |
| Vesicular quality more marked in no case. | Vesicular quality more marked in 14 cases. |
| Higher pitch of sound in 12 of 19 examinations. | Higher pitch of sound in no case. |

EXPIRATORY SOUND.

| *Right.* | *Left.* |
|---|---|
| Present on this side, and not on left side, in 3 cases. | Present on this side, and not on right side, in no case. |
| More intense on this side in 2 cases. | More intense on this side in no case. |
| Prolonged in several cases. | Prolonged in none. |
| An interval between the sounds of inspiration and expiration in several cases. | The two sounds continuous. |
| Pitch higher than that of the inspiratory sound in 11 instances. | Pitch higher in 1 instance. |
| Pitch lower than that of inspiration in 4 instances. | Pitch lower in 10 instances. |

The resonance, further, of the *voice* was noted in the same region as greater on the right side in 19 out of 23 examinations.

In looking over the pages treating of Auscultation in Disease, we observe that the term bronchial-respiration is made to embrace the bronchial and blowing respiration of Walshe. Rude respiration, which of all modifications of the respiratory murmur is the most difficult to determine, is, we think, very happily described as broncho-vesicular respiration. Dr. Flint sums up its character as "*Inspiration* presenting vesicular and tubular qualities mixed; shortened in duration; pitch raised; intensity variable; sometimes alone present. *Expiration*, oftener present; frequently existing alone; prolonged; occurring after an interval; pitch higher than that of inspiration and oftener more intense."

The difference between bronchial and cavernous, denied by some of the best auscultators of the day, consists, according to Dr. Flint, in the blowing character, low pitch and slow evolution of the sound; also in its feeble expiration, the pitch of which is even lower than that of inspiration.

The râles are described as bronchial, vesicular, cavernous, and indeterminate. In the bronchial râles, why distinguish between fine mucous râle and subcrepitant? Where is the line of division to be drawn? Again, we do not see why, in the table exhibiting the diagnostic indications of the different râles, mucous râles confined to one side of the chest should be indicative of secondary bronchitis, an affection frequently met with in typhus and typhoid fever, and with râles (as often dry as moist) as much diffused over the chest, as in primary bronchitis. We have turned to the chapter on Secondary Bronchitis for information, but meet with no further explanation of the statement.

The phenomena of the *voice* are described under four heads, Exaggerated, Vocal, Resonance and Bronchophony; diminished and suppressed vocal resonance, pectoriloquy including amphoric voice and egophony. This last named sign Dr. Flint regards as rare: Pectoriloquy is used under the signification of transmitted voice, without any reference to cavities: in the Appendix it is stated, that if in "whispering" pectoriloquy the souffle accompanying the act of whispering be of a low pitch, this is indicative of a cavity.

The theory of Skoda, with regard to the transmission of the voice being dependant upon consonance, is decidedly rejected. The arguments advanced are mainly the same as those in the second edition of Walshe, p. 142, *et seq.*

In part II, which embraces nearly 300 pages, the separate diseases are considered. In this part we confess, at first sight, to have been somewhat disappointed. It seemed to us, in many respects, as more of a compilation of the opinions of others than the first part, but it gains on repeated perusal. The remarks on diagnosis are mostly characterized by care, and evidently worked out *con amore.* We shall only notice some of the more salient points, and refer the reader to the work itself for a description of the individual diseases.

Acute lobar-pneumonia, or *Pneumonitis*, presents marked physical signs, by which its different stages can be detected. On this point observers agree; but with regard to the relative frequency and importance attached to separate signs, opinions are not as unanimous. The crepitant râle was considered by Laennec as pathognomic of the first stage; many of the more recent clinical teachers, Chomel, Cruvelhier, Skoda, deny its frequency and pathognomic import. Grisolle, on the other hand, in 373 cases of acute pneumonia, only found it altogether absent four times. Flint states, that out of 44 cases, a crepitant râle was marked in 32; in twelve its presence was not noted. This difference in opinion may, we think, be reconciled by assuming, that nearly each physician has formed his own type of a crepitant râle. Some adhere strictly to the description of the râle as given by Laennec, others include more or less all the finer bubbling sound under the name crepitant. Skoda admits as much when he says, (Am. edit. p. 157.) "moreover, I have not only not found this crepitation constantly present in pneumonia, but if we are to follow Laennec's description of it *very closely*, (the italics are our own,) I must say that I have not often observed it."

. In the second stage, or that of consolidation of tissue, dulness on percussion, bronchial respiration, and broncophony are generally met with. In two out of twenty-seven cases observed by Dr. Flint, words were transmitted (pectoriloquy); in several instances (contrary to the opinion expressed by Walshe) "whispering voice" was observed. On the puff or *souffle*, accompanying the act of whispering ("whispering bronchophony,") Dr. Flint lays considerable stress. It is generally of an acute and high pitch, and constitutes an important sign of solidification. He also notices the line of demarcation between the healthy and solidified lung, which if the inflammation, as it generally is, be confined to one lobe, will be found "in a direction coincident with the inter-lobular fissures." We are not aware of this fact having been elsewhere noticed.

"Thus if the lower lobe be affected, the line intersecting the several points at which the change in the percussion-sound is observed, extends obliquely upward and outward, from between the fifth and sixth ribs, in a direction towards the vertebral extremity of the spinous ridge of the scapula,—this being the situation of the fissure separating the upper and lower lobes on the left side, and the middle and lower lobes on the right side. On the right side, in cases in which the inflammation extends to the middle lobe, the line pursues a direction upward and outward from the fourth cartilage. This is a point not only of interest, but one which may be in some instances of improvement in diagnosis. In the absence of the auscultatory phenomena distinctive of solidification of lung, which, although generally present, may yet be absent, the question will perhaps arise whether marked dulness or flatness on percussion be not due to liquid effusion; in other words, the differential diagnosis between pneumonitis and pleuritis is to be made. Now, if under these ciscumstances, the line denoting the limits of the dulness and flatness be found to occupy the situation of the interlobular fissure, while the body of the patient is in a vertical position, the question may be considered almost or quite settled." (p. 407.)

We shall not enter into a synopsis of the chapter on *Pulmonary Tuberculosis.* The physical signs are mainly illustrated from an analysis of one hundred cases; the dulness in most was at the apex, and sufficiently well-marked. Yet even with complete and considerable solidification at the summit of the chest, an abnormal clearness was occasionally noticed. It is extremely difficult to explain this apparent anomaly, excepting, perhaps, by adopting Skoda's theory with regard to the production of tympanitic sound. (Op. cit. p. 47.) In appreciating the relative amount of dulness, the importance of the observations, already quoted, on the disparity in the character of the percussion sounds between the two sides of the chest in healthy persons, becomes apparent.

Pulmonary Œdema; Gangrene of the Lung; Pulmonary Apoplexy; Cancer of the Lungs; Cancer in the Mediastinum, are next discussed. We find here nothing new or deserving of special notice.

*Pleurisy*, and the diseases connected with it, have evidently been studied with much care: especially may this be said of chronic pleurisy, on which Dr. Flint is the author of a valuable monograph. The signs of an effusion in acute pleurisy are mostly well-marked, and the affection presents but little difficulty in diagnosis. Flatness on percussion, absent voice, and vocal fremitus, feeble or absent respiration, constitute an almost pathognomic series of physical signs. Above the effusion the percussion sound is very clear, even tympanitic. Dilatation of the affected side, and displacement of the viscera and of the diaphragm, are secondary consequences; to these Dr. Flint adds a sense of fluctuation appreciable by palpation.

Yet all cases do not prevent these marked signs; in some—fortunately rare cases—a bronchial respiration is heard all over the lung, as in consolidation, whilst the voice, instead of being absent, is transmitted to the ear. The diagnosis of pleuritic effusion becomes now of greater difficulty. The vocal fremitus is is too unreliable to be trusted. The general symptoms, the mensuration of the chest, with a careful comparison of the sounds in the upper portion elicited by percussion, have more especially to guide us to a conclusion. Dr. Flint mentions the occurrence of these exceptional variations, but merely says that attention will in all such instances develop ample data for the discrimination.

The symptoms of chronic pleurisy are generally slightly increased respiration, night-sweats, pulse more or less accelerated, and in a large proportion of cases not any great loss of weight or emaciation. The strength is sometimes preserved in an astonishing degree. None of these symptoms are conclusive without the physical signs, as the affection sometimes occurs in a very latent manner, and is frequently mistaken for affections of other organs. In Dr. Flint's Clinical Report on Chronic Pleurisy, Buffalo, 1853, the errors of diagnosis in 18 cases are numerically stated. In four the disease had been treated as latent intermittent fever; in four the disease was supposed to be phthisis; in three the patients were presumed to be laboring under continued fevers; whilst the other seven cases divided amongst them the diagnosis of disease of the heart, liver complaint, hepatization of the lung, general debility, etc. (See op. cit. p. 26.) In this disease, therefore, physical signs become of paramount interest. Now the physical signs are mainly those of acute pleurisy in the stage of effusion; the flatness generally extends from the bone of the chest upwards; the voice and the sound of respiration are absent. Whether the effused fluid be pus (empyema) or serum cannot clinically be ascertained.

To determine if chronic pleurisy have existed at any previous period, may become a question of practical importance. On this subject, the observations in the volume before us are of the highest interest and novelty, and the reader must pardon us for reproducing the entire passage in which the characters involved in the "retrospective diagnosis" are noted. The results were arrived at by the examination of fifteen patients, at periods varying from ten months to ten years from the date of the attack, and are thus summed up:

"Diminished width of the chest, apparent on inspection in the great majority of cases. Depression or flattening at the summit of the affected side, almost invariably observed; but occasionally enlargement, which probably denotes abnormal dilatation of the cells, or emphysema. The reduction in size also shown by mensuration. The shoulder generally depressed; but in some instances this is not apparent, and it may be even raised above the level of that on the opposite side. The nipple usually depressed, but not invariably, and nearer the median line. The lower ribs converging, sometimes almost overlapping; the upper ribs diverging. The distance from the posterior margin of the scapula to the spinal column lessened, often in a notable degree; an exception to this rule obtaining, in some instances, when lateral curvature of the spine takes place, the concavity looking towards the affected side. Projection of the lower portion of the scapula, occurring in a certain proportion of instances; and, also, depression of the inferior angle below the level of that on the opposite side. The respiratory movements almost uniformly diminished in a degree more or less marked; the expansibility on the opposite side being, at the same time, exaggerated. Comparative dulness on percussion; the contrast rendered more striking by the great clearness of the percussion-resonance on the opposite side. A vesiculo-tympanitic resonance at the summit, conjoined with enlargement, denoting the supervention of emphysema. Feebleness of respiratory sound over the entire side, with few exceptions; and on the opposite side, an unusually intense vesicular murmur. A bronchial respiration sometimes observed in the interscapular space, and in other parts of the side. In the latter, especially if associated with bronchophony, probably denoting dilatation of the bronchial tubes. The respiration, in a certain proportion of cases, broncho-vesicular. The vocal resonance sometimes greater, but not uniformly. The same remark applicable to fremitus. Curvature of the spine in some cases, the inclination usually lateral, the concavity toward the affected side. The position of the heart frequently normal, but in some instances displacement of this organ; it being found to the left of its natural position and elevated, if the pleuritis be seated in the left side.

"It will be borne in mind that this summary embraces characters observed in persons after complete recovery from chronic pleuritis, and presumed to be entirely free from any existing pulmonary disease, excepting, in some instances, emphysema and dilatation of the bronchial tubes." (p. 577.)

Remarks on Pneumothorax, Pleurodynia ("Pleuralgia") and Foreign Bodies in the Air-passages conclude a work, of which we cannot but admire the spirit that has presided over its composition. There is an evident accuracy aimed at throughout, by means of the carefully noted cases, and a searching after truth which recommends the volume highly to the attention of the profession. On some subjects Dr. Flint's subsequent *data* may lead him perhaps to modify some of his observations, or to substitute for the descriptions of others his own experience; but the whole character of the work is such, that it cannot fail to raise him high in the opinion of his medical brethren, as an exact and most conscientious observer.

---

*Transactions of the American Medical Association.* Vol. viii., 1855.

*The Report of the Commitee on the Hygrometrical state of the Atmosphere in various localities, and its influence on Health.* By Sanford B. Hunt, M. D.

The effect of the different states of the atmosphere upon the health of man is a problem daily increasing in interest, not only to the scientific medical practitioner, but to all who desire to increase the happiness of the human race. It is well known that the tendency to certain diseases is far greater in some seasons than in others; and the inquiry has often been made whether the extraordinary prevalence of those diseases is owing to the state of the atmosphere alone, or to what has been called "*terrene causes*," such as filth, exhalations from decaying vegetable matter, or stagnant water, or to both of these causes combined. That such an inquiry possesses considerable interest, we are all ready to acknowledge. It is interesting, not only as leading to the discovery of a long hidden truth, but also in its results; for if the true causes of those diseases can be clearly ascertained, they may, to a great extent, be prevented, or at least ameliorated in their effects. If owing to the state of the atmosphere, we may to a certain extent guard against its effects; if to terrene causes, an active sanitary police may prevent their existence, or remove them as soon as they are discovered. In the prosecution of this inquiry, however, we are hindered by the extreme complexity of the elements, and the difficulty of arriving at an accurate or reliable knowledge of their effects. We know not whether the effects of the weather should be attributed to the pressure of the atmosphere, the influence of prevailing winds, the heat, dryness or humidity of the air, or to all of them combined. Besides, the science of meteorology is still in its infancy; its literature is scattered and imperfect, and even the method of ascertaining the most essential conditions are yet unsettled, different methods being in use by different observers.

These difficulties being obvious to the members of the American Medical Association, they acted wisely in endeavoring to isolate one atmospherical condition, and to trace its effects, first, separately, and then when combined with others acting with and upon each other and upon the health and life of man. The subject of the "Influence of the Hygrometrical condition of the Atmosphere on Health," was accordingly assigned to Dr. Hunt, of Buffalo, by whom the Report before us was made.

In this paper the subject of Humidity in connection with heat, and its effects when combined with other causes, in inducing certain diseases, is very forcibly, clearly and logically treated, and in many of its results illustrated by facts occurring under the observation of the author.

He begins by asserting that, "in all calculations of the humidity of the atmosphere, the *dew-point* is the essential condition, as it indicates the point to which the atmosphere is saturated with moisture, all between that and the temperature being a margin for the absorption of vapor." The method of ascertaining the dew-point is next explained, and some of the most important causes of its variation pointed out. Attention is next directed to the relative humidity of different regions of the United States, and the conclusion reached, that the greatest "humidity will be formed at low altitudes open to south-west winds;" and that "the relative humidity of the atmosphere will decrease with the increasing altitude of the place examined, with its protection from the south-west wind, and with its exposure to polar breezes. These are the great conditions which apply to all parts of our country. Disturbing local causes will vary, while they confirm the exactness of the rule."

Having established the general laws of *humidity*, as they are found to exist in different parts of the United States, he next proceeds to trace the connection between a *deficiency* or *excess* of humidity upon the health of an individual. In all his reasoning he very properly considers the *fraction* of *saturation*, or the fraction which the moisture existing in the atmosphere bears to that which would constitute saturation, as the essential condition. He shows that it is not probable that a mere deficiency of humidity can exert any direct, or, at least, immediate effect upon health; and that probably the mere fact of a high dew-point is not sufficient to cause disease, if the fraction of saturation be low, and a wide margin consequently left for evaporation. The effect of a high dew-point combined with a high fraction of saturation is next considered, and shown to be sufficient to cause "certain diseases marked by simple loss of tone, but is not sufficient *of itself* to produce zymotic or epidemic diseases." "But it is nevertheless very certain, that the combination of high heat and humidity is one of a series of causes, having a direct influence upon the prevalence and fatality of epidemics when once established. The *inception* is due to a special cause, of which we are ignorant; its *progress* depends largely upon the relative humidity of the air." When we find a *high dew-point* combined with *terrene causes*, such as filth, or some upheaval of the soil, cleanly or dirty, then the cholera or yellow fever follows. One of these, the terrene causes, for example, may exist without epidemic disease, but its development into an actual epidemic will take place as soon as a high dew-point is supervened. The two causes, in order to produce the dreaded effect, must act together. In fine, Dr. Hunt concludes his most valuable paper by summing up his reasoning in the following words :

"It is proven that certain epidemic diseases are materially modified by *ærial* causes ; that these causes alone are not sufficient for the actual production of true zymotic disease, or any other effect save nervous exhaustion ; that, combined with the *terrene* conditions, they exert a direct, marked and fatal influence upon epidemics, and that these causes are separable ; the the terrene condition being removable by the judicious exercise of the sanitary police, and the ærial causes being avoidable by hygienic measures, based upon a knowledge of the habitudes of aqueous vapor at ordinary temperatures."

Our deep interest in a subject which may in a few months be of such vital importance, has already led us to exceed our limits, and we must conclude by recommending to the attention of all this intensely interesting and important paper.

---

*A Practical Hand-book of Medical Chemistry.* By JOHN E. BOWMAN, F. C. S., Professor of Practical Chemistry in King's College, London. *Second American from the Third and Revised Landon Edition. With Illustrations.* Philada. Blanchard & Lea. 1856.

This admirable little work cannot but prove a valuable guide to all who desire instruction in the proper mode of analysing the blood, urine and other products of the human body. Practical directions for detecting poisons in organic mixtures and in the tissues are also given, which will be found very useful to all who desire information on such matters. All the recent discoveries in medical chemistry have been added to the present edition, thus placing it in advance of the previous works upon the same subject.

*The Principles of Surgery.* By JAMES MILLER, F. R. S. E., F. R. C. S. E., &c. *Fourth American from the Third and Revised English Edition. Illustrated by Two Hundred and Forty Engravings on wood.* Philada. Blanchard & Lea. 1856.

The high estimation in which Prof. Miller's "Principles" are held throughout England and our own country dispenses with the necessity of saying anything more of the present American edition than that it is a transcript of the third and revised English one. We can strongly recommend it to our readers as a most useful and desirable work.

